# Porous Organic Cage-Embedded C10-Modified Silica as HPLC Stationary Phase and Its Multiple Separation Functions

**DOI:** 10.3390/molecules27248895

**Published:** 2022-12-14

**Authors:** Litao Wang, Siqi Han, Haiyang Yu, Qinghua Yu, Dong Pei, Wenjing Lv, Jiasheng Wang, Xingyu Li, Ruifang Ding, Qibao Wang, Mei Lv

**Affiliations:** 1 School of Pharmacy, Jining Medical University, Jining 272000, China; 2 School of Pharmacy, Weifang Medical University, Weifang 261000, China; 3 Qingdao Center of Resource Chemistry & New Materials, Lanzhou Institute of Chemical Physics, Chinese Academy of Sciences, Qingdao 266000, China

**Keywords:** C10-embedded in organic molecular cages, stationary phase, multiple separation functions, synergy, high performance liquid chromatography

## Abstract

Reduced imine cage (RCC3) was covalently bonded to the surface of silica spheres, and then the secondary amine group of the molecular cage was embedded in non-polar C10 for modification to prepare a novel RCC3-C10@silica HPLC stationary phase with multiple separation functions. Through infrared spectroscopy, thermogravimetric analysis and nitrogen adsorption–desorption characterization, it was confirmed that RCC3-C10 was successfully bonded to the surface of silica spheres. The resolution of RCC3-C10@silica in reversed-phase separation mode is as high as 2.95, 3.73, 3.27 and 4.09 for p-phenethyl alcohol, 1-phenyl-2-propanol, p-methylphenethyl alcohol and 1-phenyl-1-propanol, indicating that the stationary phase has excellent chiral resolution performance. In reversed-phase and hydrophilic separation modes, RCC3-C10@silica realized the separation and analysis of a total of 70 compounds in 8 classes of Tanaka mixtures, alkylbenzene rings, polyphenyl rings, phenols, anilines, sulfonamides, nucleosides and flavonoids, and the analysis of a variety of chiral and achiral complex mixtures have been completed at the same time. Compared with the traditional C18 commercial column, RCC3-C10@silica exhibits better chromatographic separation selectivity, aromatic selectivity and polar selectivity. The multifunctional separation mechanism exhibited by the stationary phase originates from various synergistic effects such as hydrophobic interaction, π-π interaction, hydrogen bonding and steric interaction provided by RCC3 and C10 groups. This work provides flexible selectivity and application prospects for novel multi-separation functional chromatographic columns.

## 1. Introduction

High performance liquid chromatography (HPLC) is a commonly used chromatographic analysis technique for qualitative and quantitative analysis of compounds. It is widely used in the analysis of pharmaceuticals, food and environmental samples due to its high separation efficiency, high accuracy and high precision [1,2,3]. The research of HPLC stationary phase has always been the focus of chromatographic separation and analysis technology, and different chromatographic columns are usually selected according to the physical and chemical properties of the analytes. For example, reversed-phase columns are the most common and are suitable for the analysis of moderately polar compounds [4]. Chiral columns are apt for the separation of specific chiral compounds [5,6,7]. In modern high performance liquid chromatography, the separation effect is largely determined by the choice of stationary phase. Therefore, the development of multifunctional stationary phases with multi-selective separation and high separation efficiency is still an important research direction in the separation discipline.

Porous organic molecular cages (POCs) are a unique class of porous materials composed of covalently bound discrete molecules with inherent cavities accessible to guest molecules [8,9]. The discrete nature of POCs makes them processable and can be modified by their skeleton structure to fabricate novel composites [10,11]. Likewise, due to the lack of intermolecular covalent bonds in POCs, porous molecules exhibit structural fluidity, which allows for cooperative interactions between host and guest [12]. Based on the excellent properties of POCs, it is easy to combine these cage-like molecules on various substrates to make materials such as adsorption separation, molecular sensing and catalysis [13,14]. At present, POCs (CC3, CC5, CC9, CC10, etc.) have been coated on capillary columns for gas chromatography by static methods, and the prepared columns show excellent enantioselectivity in the resolution of racemic compounds with baseline separation at high resolution values for most enantiomers [15,16,17,18,19,20]. In contrast, there are few reports on the preparation of HPLC stationary phases through bonding molecular cages to the surface of silica gel and their separation and analysis performance [21]. In addition, the imino groups and secondary amine groups of the molecular cage have poor stability under the acid-base conditions of the mobile phase, and there are few types of compounds to be separated. In order to give full play to the advantages of molecular cages in the application of HPLC separation and analysis, it is necessary to transform and modify the skeleton structure of molecular cages.

Molecular cage CC3 is connected by an imine formed by one-step [4+6] condensation of 1,3,5-triformyl benzene and (R,R) or (S,S)-1,2-diaminocyclohexane [22]. The imine bond in the CC3 cage can be reduced to a more stable secondary amine group, the reduced imine cage (RCC3). The secondary amine group in the RCC3 structure can selectively undergo hydrophobic modification of the C10 group to introduce an alkyl functional group into the RCC3 structure. RCC3 not only retains the cage-like structure of the parent imine, but also contains more stable C10, tertiary amine groups and chiral pore windows in the RCC3 structure. The analyte can bind to chiral molecules with high affinity to form a chiral microenvironment, which plays an important role in the separation of chiral compounds [23,24,25,26]. In addition, the C10, benzene ring, cyclohexane, tertiary amino group and alcoholic hydroxyl group in the molecular cage structure provide hydrophobic interaction, π-π stacking interaction, hydrogen bonding interaction and hydrophilic interaction, which play an important role in the separations of various types of compounds. Therefore, these functional groups will endow RCC3 with good separation performance for various types of compounds during HPLC separation.

Herein, C10-modified RCC3 was covalently bound to the surface of silica spheres to prepare a multi-functional, high-performance, liquid chromatography stationary phase (RCC3-C10@silica). The enantiomers of phenethyl alcohol, 1-phenyl-2-propanol, p-methyl phenethyl alcohol and 1-phenyl-1-propanol were selected to investigate the chiral separation performance of the stationary phase. Taking commercial WondaSil^®^ C18-WR column as a reference, Tanaka mixtures, alkylbenzenes, polyphenylene rings (PAHs), phenols, anilines, sulfonamides, nucleosides and flavonoids were selected as analytes. The separation and analysis performance of the stationary phase was investigated in reversed phase liquid chromatography (RPLC) mode. The separation performance of a variety of chiral and achiral complex mixtures was also investigated. This work provides a reference idea for the development of new multi-functional HPLC stationary phase materials. 

## 2. Results and Discussion

### 2.1. Characterization of RCC3-C10@silica

The chemical composition of the stationary phase was characterized by FTIR. The FTIR spectra of activated silica (a), RCC3@silica (b) and RCC3-C10@silica (c) were shown in Figure S2. Compared with band (a), the trace infrared absorption peak of band (b) at 1623 cm^−1^ (secondary amine group in the molecular cage) indicates that RCC3 is covalently attached to the silanol group. The enhanced peaks at 2938 and 2867 cm^−1^ can be attributed to the stretching vibration of the methylene group (CH_2_) in the molecular cage [27]. The reduced peak at 1623 cm^−1^ in band (c), while there is increased peak intensity at 2938 cm^−1^ and 2867 cm^−1^, is due to the introduction of C10 chains, which confirms the success of the RCC3-C10@silica stationary phase preparation. 

The organic content of the RCC3-C10@silica stationary phase was characterized by TGA, DSC and DTG analysis, and the results are shown in Figure S3. It can be seen from the TGA curve that the weight loss value of the stationary phase increases gradually during the gradual heating process. The weight loss of the stationary phase below 100 °C was caused by the evaporation of the water adsorbed on the surface silica gel. When the temperature reached above 100 °C, the organic components of the RCC3@silica stationary phase began to decompose and reached a stable equilibrium at 730 °C, and the mass loss was about 36%, which indicates that the loading of the RCC3-C10 molecular cage is about 36%. From the DSC curve, it can be seen that the weight loss process continues to be endothermic at the temperature of 223 °C. Combined with the TGA curve, it can be seen that the RCC3-C10 molecular cage bonded to the surface of the silica gel is endothermic during the thermal decomposition process. It can be seen from the DTG curve that the weight loss of the stationary phase during the heating process was divided into two stages. The mass losses were 0.54 g and 0.13 g at 222–352 °C and 456–594 °C, respectively. It is speculated that the two parts of the mass loss may be the RCC3 molecular cage and the C10 group, respectively, which further proves the successful preparation of the RCC3-C10@silica stationary phase.

Nitrogen adsorption and desorption experiments evaluated the specific surface area and porosity of silica gel and RCC3-C10@silica (Figure S4). As can be seen from Figure S4a, the isotherm of silica gel belongs to the IV type in the IUPAC classification, the H1 hysteresis loop. In the low-pressure section (P/P_0_ = 0.0–0.1), the amount of adsorption increases gently, and N_2_ molecules are adsorbed on the inner surface of the mesopores in a single- to multi-layered manner. There is a sudden increase in the adsorption capacity around P/P_0_ = 0.65–0.9. This area reflects the size of the pore size of the sample, and its variation width can be used as a basis for measuring the homogeneity of the mesopores. Its specific surface area can be determined from nitrogen adsorption and desorption isotherms to be 344 m^2^/g, and the average pore size distribution is 7.3 nm. It can be seen that the isotherm of RCC3-C10@silica belongs to type IV in the IUPAC classification from Figure S4b, H2 hysteresis loop, and the reason for the H2 hysteresis loop may be porous adsorbate or uniform particles (RCC3-C10) caused by stacking holes. There is a sudden increase in the adsorption capacity around P/P0 = 0.5–0.8, which may be due to the occurrence of capillary condensation. Its specific surface area was 211 m^2^/g, and the average pore size distribution was 3.5 nm as determined by nitrogen adsorption and desorption isotherms. After the stationary phase was bonded to the silica gel, the specific surface and pore size distribution were reduced, indicating that the bonding between the RCC3-C10 molecular cage and the silica gel occurred on the inner surface of the silica gel pores, thus proving that the stationary phase was successfully bonded.

### 2.2. The Evaluation of Chromatographic Separation Performance 

#### 2.2.1. The Resolution of Chiral Alcohols

**Figure 1:**
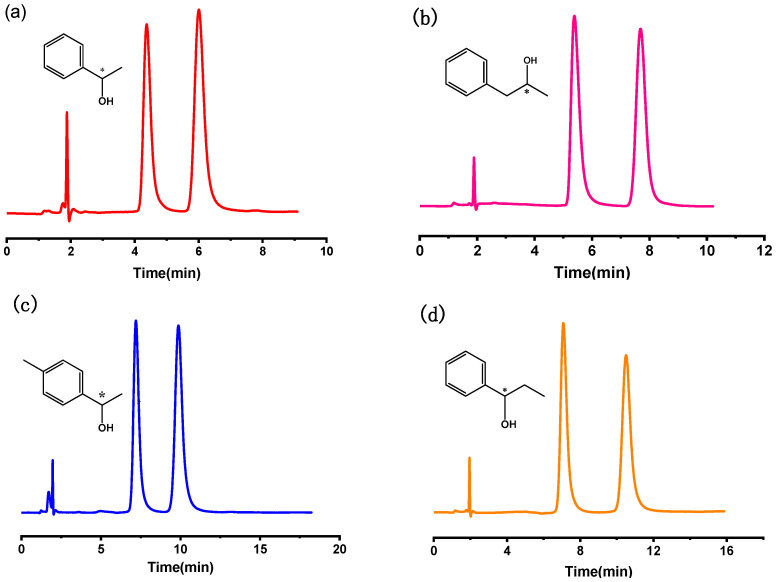
Chromatogram of (**a**) phenyl ethanol, (**b**) 1-phenyl-2-propanol, (**c**) p-methyl phenyl-ethanol, (**d**) 1-phenyl-1-propanol on the RCC3-C10@silica molecular cage column. Mobile phase: 40% methanol/water (*v*/*v*); Flow rate: 1.0 mL/min; UV: 254 nm.

At present, chiral drugs occupy most of the market in drug production, but different enantiomers of chiral drugs have different pharmacological activities, so the separation of chiral drugs is very important. Four chiral compounds, phenethyl alcohol, p-methyl phenethyl alcohol, 1-phenyl-1-propanol and 1-phenyl-2-propanol, were selected to test the resolving power of the molecular cage column for chiral compounds. As shown in Figure 1, the chromatographic parameters of the chiral compounds on the molecular cage column are listed in Table 1. The four chiral compounds were all separated by the baseline under the mobile phase condition of 40% methanol and water, and the resolutions were 2.95, 3.73, 3.27 and 4.09, respectively. The molecular structures of these four chiral compounds all contain hydrogen bond donor groups. The benzene ring and hydroxyl group in the molecular structure of chiral alcohol analytes can have hydrogen bonding, π-π interaction, hydrophobic interaction and the synergistic interaction of various forces with the functional groups of the molecular cage. This may be one of the reasons for the efficient resolution of chiral alcohol compounds. In addition, the chiral microenvironment formed by the inclusion between host and guest may also be the main reason for the separation of chiral compounds.

#### 2.2.2. Tanaka Test Analysis

Tanaka test can be used to evaluate chromatographic separation performance in RPLC mode [28]. Caffeine, uracil, phenol, butylbenzene, pentylbenzene, o-terphenyl and triphenylene were separated on the RCC3-C10@silica molecular cage column and C18 commercial column with 80% methanol as mobile phase, and the chromatographic separation was shown in Figure S5. The retention factor (k) and selection factor (α) of stationary phases were summarized in Table S1. The RCC3-C10@silica molecular cage column exhibited a slightly lower k value (k of amylbenzene) than the commercial C18 column. The selection factors of butylbenzene and pentylbenzene can be used to investigate the hydrophobic selectivity of the molecular cage column. Compared with the commercial column (αpentylbenzene/butylbenzene = 1.47), the molecular cage column (αpentylbenzene/butylbenzene = 1.33) obtained slightly lower hydrophobic selectivity, which may be due to the effect of the introduced C10 chains and polyphenyl rings in the molecular cage. The results showed that the hydrophobicity of the RCC3-C10@silica molecular cage column was weaker than that of the C18 commercial column. The α of o-terphenyl and triphenylene can be used to examine the shape selectivity, α-triphenylene/o-terphenyl = 8.58, compared with the commercial column (α-triphenylene/o-terphenyl = 4.85), the RCC3-C10@silica columns have high shape selectivity, which may be attributed to the π-π interaction between the choice of test compounds and the benzene ring on RCC3, and the stereostructure of RCC3 may also play an important role in improving the shape selectivity.

#### 2.2.3. Separation of Alkyl Benzenes

Hydrophobic selectivity or methylene selectivity refers to the relative retention value of homologues that differ by one methylene group on the stationary phase. The weaker the polarity of the solute, the stronger the hydrophobicity, and the longer the retention time in the stationary phase, the higher the retention value. Taking the log k of each analyte as the ordinate and the methylene number as the abscissa to draw a straight line (Figure S6), it can be seen that each analyte has a good correlation under different mobile phase ratios. It shows that the molecular cage column has good hydrophobic selectivity.

The chromatographic separation of alkyl benzene ring homolog by the molecular cage column and C18 commercial column is shown in Figure S7. It can be seen that the benzene, toluene and other alkyl benzene homologues were separated well by both the molecular cage column and commercial column under the condition that 70% methanol/water (*v*/*v*) is the mobile phase. 

The composition of the mobile phase and the concentration of the organic solvent will also affect the hydrophobic selectivity of the stationary phase. Two different mobile phases, methanol and acetonitrile, were selected in the separation of alkylbenzene compounds to investigate the effect of organic solvents on the separation of compounds.

Take the log k of each analyte as the ordinate and the concentration of the organic solvent as the abscissa, as shown in Figure S8. When the mobile phase composition is methanol/water or acetonitrile/water, the log k increases with the length of the carbon chain on the alkylbenzene. The lower the methanol or acetonitrile content in the mobile phase, the higher the water content and the larger the log k, which is in line with the separation principle of reversed-phase chromatography. Moreover, when acetonitrile and methanol are used as mobile phases, the retention order of each substance is also the same, and the retention is stronger in acetonitrile.

#### 2.2.4. Separation of PAHs

PAHs refer to hydrocarbons containing two or more benzene rings in the molecule, which can be divided into aromatic fused rings and aromatic non-fused rings. The fused polycyclic type has two or more shared carbon atoms between the benzene rings, such as naphthalene, anthracene and phenanthrene. PAHs mainly come from the combustion of various fuels and wastes discharged from factories such as the organic chemical industry and petroleum industry. Due to their strong carcinogenicity, wide distribution and easy accumulation in living organisms, they are very harmful to the natural environment and human health. Therefore, it is necessary to select an appropriate method for its detection.

The chromatographic separation diagram of benzene, naphthalene, acenaphthylene, anthracene, o-terphenyl, m-terphenyl and biphenyl when the analytes are under 80% methanol/water (*v*/*v*) mobile phase is shown in Figure S9. It can be seen that the seven PAH compounds were separated well on the RCC3-C10@silica column and the C18 commercial column. The elution order of the analytes is related to the number of benzene rings. The more benzene rings contained, the stronger the hydrophobicity, the higher the retention factor and the later the peaks appear. The reason is that acenaphthene has a methylene group and strong hydrophobic selectivity, which echoes the separation of alkylbenzene rings. O-terphenyl, anthracene and m-terphenyl all have three benzene rings, but due to the different connection methods of the benzene rings, the more linear the molecular structure is, the larger the retention factor will be and the later the peak will appear. Both biphenyl/acenaphthylene and o-terphenyl/para-terphenyl isomers were well separated on both the molecular cage and commercial columns probably due to steric interactions between the stationary phase and the analyte.

Anthrone, 2-methylnaphthalene, dibenz[a,h]anthracene, biphenyl, pyrene, quinone, triphenylene and p-terphenyl were selected as the analytes under 90% methanol/water (*v*/*v*) mobile phase. The chromatographic separation diagram are shown in Figure S10, and the k and α are shown in Table S2. It can be seen that these seven analytes can be well separated on the two types of chromatographic columns. The mixed samples could be completely separated by the RCC3-C10@silica column, and the elution order of the mixture was consistent with the number of additional benzene rings of PAHs. The isomer quinone/triphenylene could also be separated well, the α_triphenylene/p-terphenyl_ of the molecular cage = 2.55 > the α_triphenylene/p-terphenyl_ of WondaSil^®^ C18-WR = 1.82. Therefore, the plane selectivity of the RCC3-C10@silica column was slightly higher than that of the C18 commercial column.

#### 2.2.5. Separation of Polar Aromatic Analytes

**Figure 2:**
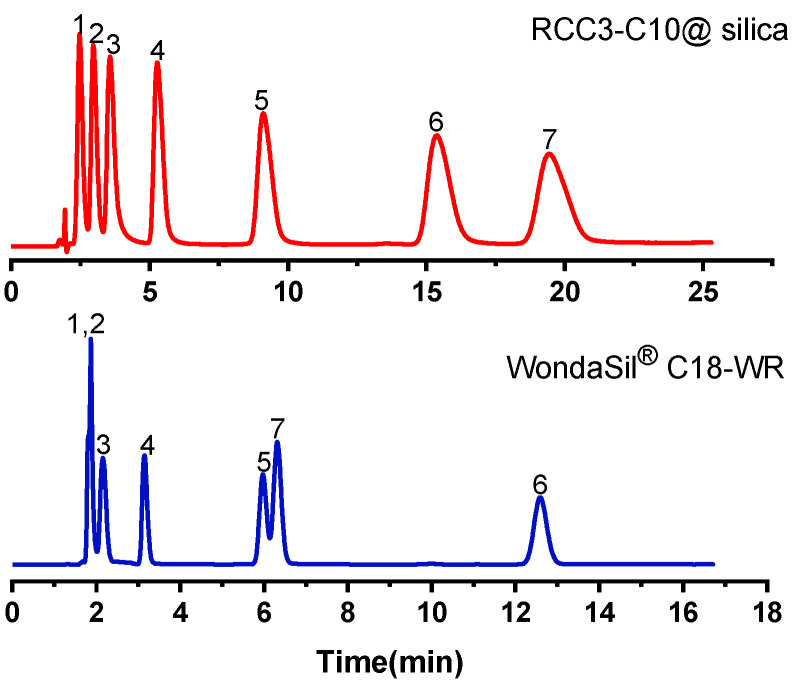
Chromatograms of phenol compounds on the RCC3-C10@silica column and WondaSil^®^ C18-WR column. Analytes: (1) p-aminophenol, (2) m-aminophenol, (3) o-aminophenol, (4) phenol, (5) 3, 5-dimethyl phenol, (6) p-tert-butyl phenol, (7) 2-naphthol. Mobile phase: 60% methanol/water (*v*/*v*); Flow rate: 1.0 mL/min; UV: 234 nm.

Since the cage molecule has a benzene ring structure, when separating aromatic compounds, the compound and the cage molecule will have π-π interaction, and the polar group in the polar compound will also have hydrogen bond interaction with the amino group in the molecular cage. Phenols and anilines were selected to test the separation performance of RCC3-C10@silica column for polar aromatic compounds. The chromatographic separation diagrams of phenolic compounds under the chromatographic conditions of 60% methanol/water (*v*/*v*) are shown in Figure 2 and Figure S11. The k and α of phenolic compounds on the RCC3-C10@silica column and C18 commercial column are shown in Table S3. Both the isomers p/m-catechol and p/m/o-aminophenol were well separated on the RCC3-C10@silica column. This indicates that for the separation of phenol compounds, in addition to the hydrophobic interaction, the π-π interaction and hydrogen bonding between the stationary phase and the analytes can also promote the identification and separation of isomers. The tert-butylphenol appeared faster than 2-naphthol on the RCC3-C10@silica column, which further confirmed the existence of multiple interaction mechanisms between the RCC3-C10@silica column and the compounds.

**Figure 3:**
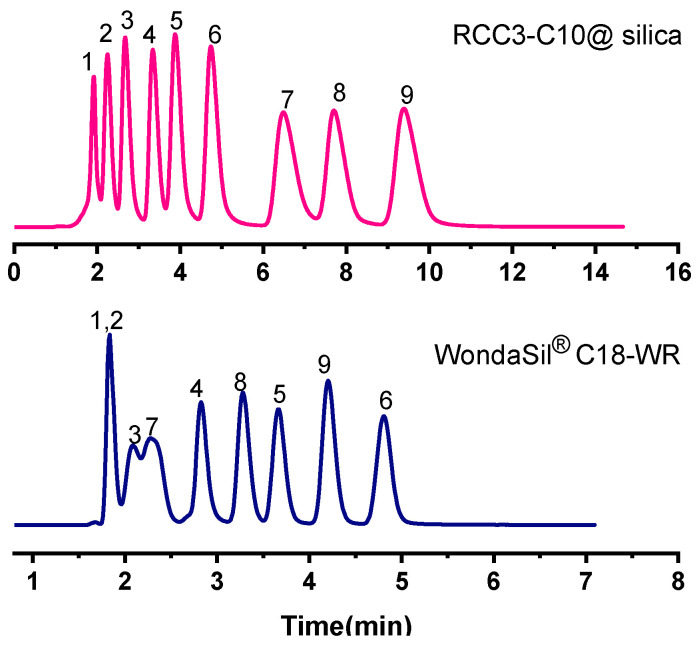
Chromatograms of anilines on the RCC3-C10@silica column and WondaSil^®^ C18-WR column. Analytes: (1) p-phenylenediamine, (2) m-phenylenediamine, (3) o-phenylenediamine, (4) aniline, (5) p-toluidine, (6) 3, 4-dimethylamine, (7) 4, 4-biphenylenediamine, (8) m-nitroaniline, (9) o-nitroaniline. Mobile phase: 60% methanol/water (*v*/*v*); Flow rate: 1.0 mL/min; UV: 254 nm.

The chromatographic separation of anilines under 60% methanol/water (*v*/*v*) chromatographic conditions is shown in Figure 3, and the k and α of anilines on the RCC3-C10@silica column and C18 commercial column are shown in Table S3. The anilines could achieve better separation on the RCC3-C10@silica column, and the peak shape was well symmetrical. On the C18 commercial column, the separation of 4,4-benzidinediamine and o-phenylenediamine could not be achieved because the separation of the two could not be achieved by the single hydrophobic interaction on the C18 commercial column. However, the π-π interaction and hydrogen bonding provided by the molecular cage group may be the main reason for the late emergence of 4,4-benzidinediamine on the RCC3-C10@silica column. There are various synergistic effects such as hydrogen bonding and π-π between nitroaniline and the molecular cage stationary phase, which makes the retention on the molecular cage’s stationary phase stronger.

#### 2.2.6. Separation of Nucleosides and Sulfonamides

Four hydrophilic nucleosides, cytidine, thymidine, guanosine and inosine, were selected and separated on the RCC3-C10@silica column and C18 commercial column as shown in Figure S12. On the molecular cage column, due to the existence of RCC3, there are various separation mechanisms, such as hydrogen bonding and π-π interaction between the molecular cage and the compound, so that these four nucleosides can be well separated on the RCC3-C10@silica column. 

The five sulfonamides were well resolved on the RCC3-C10@silica column (Figure S13). The sulfathiazole remained the strongest because there were more hydrogen bond acceptors in the sulfathiazole structure, and it was more closely combined with the molecular cage immobilization phase. Its peak retention time was the latest. In the molecular structure of sulfadiazine and sulfadiazine, the hydrophilicity of the compound decreases as the number of methyl groups increases, and the binding to the hydrophilic stationary phase is weakened; thus, the peak time of sulfadiazine was later than that of sulfadiazine. However, the peak time of sulfamethazine was earlier than that of sulfamethazine because sulfamethazine has strong hydrophobicity, is closely combined with the molecular cage immobilization phase and has a stronger retention effect. This further showed that the separation mechanism of the molecular cage columns was complex, and there are multiple mechanisms of interactions between molecular cage and analytes which could be used for the separation and analysis of hydrophilic compounds (such as nucleosides and sulfonamides).

#### 2.2.7. Separation of Flavonoids

Flavonoids are natural active ingredients present in plants and have a very wide range of medicinal values. In the process of separation and analysis of flavonoids as natural medicines, modern HPLC plays a very important role in the identification process of flavonoids. Figure S14 showed the separation chromatograms of four flavonoids under the chromatographic conditions of 70% methanol/water (*v*/*v*). The flavanones were well resolved on both the RCC3-C10@silica column and the C18 commercial column, but the retention order was completely opposite, which may be attributed to the hydrogen bonding and π-π interactions provided by the molecular cage.

#### 2.2.8. Separation of Complex Mixture

**Figure 4:**
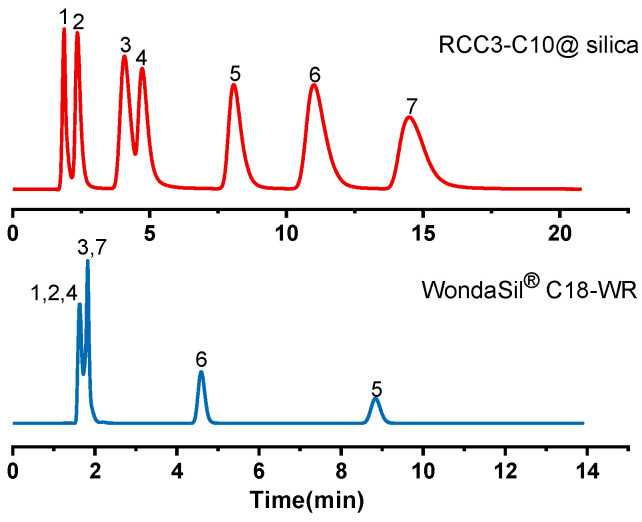
Chromatogram of nucleosides, flavonoids and sulfonamides on the RCC3-C10@silica molecular cage column and WondaSil^®^ C18-WR column. Analytes: (1) cytidine, (2) sulfanilamide, (3) naringin, (4) inosine, (5) flavanone, (6) 6-hydroxyflavanone, (7) sulfadimidine. Mobile phase: 70% methanol/water (*v*/*v*); Flow rate: 1.0 mL/min; UV: 254 nm.

**Figure 5:**
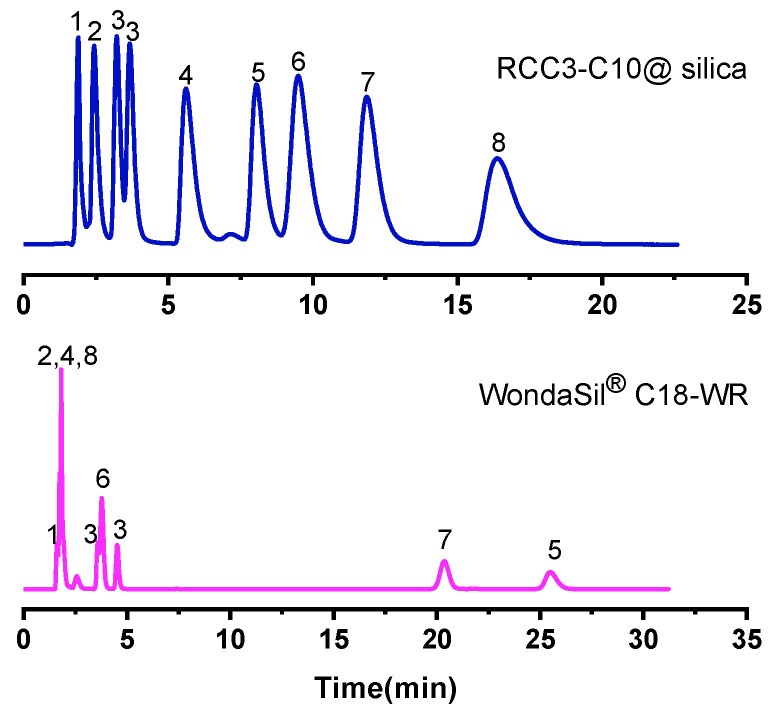
Chromatogram of chiral and achiral compounds on the RCC3-C10@silica molecular cage column and WondaSil^®^ C18-WR column. Analytes: (1) cytidine, (2) o-phenylenediamine, (3) 1-phenyl-1-propanol, (4) sulfapyridine, (5) tert-butyl benzene, (6) 4-hydroxyflavanone, (7) biphenyl, (8) sulfadimidine. Mobile phase: 70% methanol/water (*v*/*v*); Flow rate: 1.0 mL/min; UV: 254 nm.

It can be seen from the above analysis that the RCC3-C10@silica column has the characteristics of a typical RPLC. Both chiral and achiral compounds have good separation effect. In this experiment, in order to investigate whether the separation of different types of compounds will interfere with each other, nucleosides, flavonoids and sulfonamides in achiral drugs were selected as analytes. Many compounds were well separated as shown in Figure 4 and Figure 5. Among them, it can be observed that the separation of different types of compounds on the molecular cage column did not cause interference and could be well separated. The reason is that the multiple retention mechanisms based on the molecular cage column include hydrogen bonding, π-π interaction and hydrophobic interaction. The separation sequence of these compounds on the C18 column was different, possibly due to a single hydrophobic separation mechanism. In addition, when the composition of the mixture is uncertain, the use of RCC3-C10@silica column has a great advantage in separation selection and could realize the separation of complex components, which has great commercial value.

The results of the above various analytes showed that the stationary phase has good separation performance. The designed and prepared RCC3-C10@silica multifunctional chiral stationary phase contains many functional groups’ active sites and chiral pockets, which can combine with chiral molecules with high affinity to form a chiral microenvironment. Meanwhile, the chiral interaction site provides multiple selectivity for the combination of chiral compounds and stationary phases, which has excellent chiral resolution performance. More importantly, the RCC3-C10@silica stationary phase with the synergistic effect of the RCC3 and C10 chains provides multiple retention mechanisms including hydrophobic interactions, π-π interactions and hydrogen bonding, which endowed with the multiple retention mechanisms for this newly prepared chiral stationary phase. Relatively achiral compounds also showed good separation effect.

### 2.3. The Efficiency and Repeatability of RCC3-C10@silica Column

In the application process of chromatographic analysis, repetitive experiments are of great significance. RCC3-C10@silica column was made three times, and these columns are repeatable in the separation and analysis performance of various compounds. Benzene, phenol, aniline, naphthalene, flavanone, sulfonamide and guanosine were selected for repeatability evaluation, and the stability of chromatographic column separation analysis was investigated. The intraday relative standard deviation (RSD) of the analytes, calculated according to Figure S15a, fluctuated between 0.14–0.36%, and the intraday RSD of the analytes, calculated according to Figure S15b, fluctuated between 1.77–2.76%, so it can be seen that RCC3-C10@silica column has very good stability in RPLC. Benzene, naphthalene, phenol, aniline, flavanone and cytidine were selected as test samples, and the column efficiency of RCC3-C10@silica was calculated, in which the column efficiency of benzene, naphthalene, phenol, aniline, cytidine and flavanone was 16,304 N/m, 15,360 N/m, 11,985 N/m, 13,205 N/m, 9673 N/m and 7266 N/m, respectively. Overall, RCC3-C10@silica column has satisfactory efficiency and reproducibility for the separation of various compounds.

## 3. Materials and Methods 

### 3.1. Materials 

In order to prepare a stationary phase, silica (diameter: 5 μm, pore size: 100 Å, surface area: 306 m^2^ g^−1^) was purchased from Fuji Silysia Chemical Ltd. (Fuji, Aichi, Japan). 1,3,5-Triformylbenzene (purity 99%) was purchased from Beijing Yisiyan Co., Ltd., and dichloromethane was purchased from Tianjin Fuyu Co., Ltd. (Tianjin, China). Moreover, 3-(2,3-glycidoxy)propyltrimethoxysilane (purity 97%) was purchased from Adamas-beta Reagent Co., Ltd. (Shanghai, China). Flavonoids (flavanone, 6-hydroxyflavanone, 4’-hydroxyflavanone, equol) were purchased from Shanghai Merrill Chemical Technology Co., Ltd. (Shanghai, China). In addition, (1R,2R)-1,2-cyclohexanediamine (purity 99%), sulfonamides (sulfonamides, sulfadiazine, sulfapyridine, sulfathiazole, sulfamethazine, sulfamethazine), nucleosides (Cytidine, thymidine, guanosine, inosine, naringin, uracil) were purchased from Tianjin Xiens Biochemical Technology Co., Ltd. (Tianjin, China). C10-Br was purchased from Shanghai Jingpure Reagent Co., Ltd. (Shanghai, China). Chromatographic grade methanol was purchased from Shandong Yuwang Co., Ltd. (Dezhou, Shandong, China). Alkylbenzenes (toluene, ethylbenzene, propylbenzene, butylbenzene, pentylbenzene), PAHs (benzene, naphthalene, acenaphthene, anthracene, pyrene, anthrone, biphenyl, 2-methylnaphthalene, dibenz[a,h]anthracene, o-terphenyl, m-terphenyl, p-terphenyl, triphenylene, tert-butylbenzene) were purchased from Shanghai McLean Biochemical Technology Co., Ltd. (Shanghai, China). Pyridine, absolute ethanol, phenols (phenol, 2-naphthol, 3,5-dimethylphenol, o-aminophenol, m-aminophenol, p-aminophenol, p-tert-butylphenol, hydroquinone, m-phenylene Diphenol, catechol, p-cresol, p-dimethylphenol, diphenyl ether), amines (aniline, o-phenylenediamine, m-phenylenediamine, p-phenylenediamine, p-toluidine, 3,4-dimethylaniline, 4,4-benzidinediamine, o-nitroaniline, m-nitroaniline, caffeine) were purchased from Sinopharm Chemical Reagent Co., Ltd. (Shanghai, China). (±)-phenethyl alcohol and (±)-p-methylphenethyl alcohol were purchased from Guangzhou Yangye Biotechnology Co., Ltd. (Guangdong, China), and (±)-1-phenyl-2-propanol and (±)-1-phenyl-1-propanol alcohol were purchased from Huazhong Haiwei Technology Co., Ltd. (Hubei, China). WondaSil^®^ C18-WR commercial column (4.6 mm × 150 mm, 5 µm) was purchased from Shimadzu Corporation (Shimadzu, Kyoto, Japan). 

### 3.2. Apparatus

All chromatographic analyses were conducted on SHIMADZU series high performance liquid chromatography system, which consists of LC-10ATVP pump, CTO-10ASVP column oven, equipped with SIL-10AVP injection valve and SPD-M10AVP UV detector. Fourier transform infrared spectroscopy (FTIR) was obtained on an IRTracer-100 Fourier transform infrared spectrometer (Shimadzu, Kyoto, Japan). Thermogravimetric analysis (TGA), Differential Scanning Calorimetry (DSC) and Derivatives Thermogravimetry (DTG) were performed on a TGA/DSC 3+ Thermogravimetric Analyzer (Mettler Toledo, Columbus, OH, USA) at room temperature to 800 °C at a heating rate of 10 °C/min. Nitrogen adsorption and desorption were performed on an ASAP2020 nitrogen adsorption and desorption instrument (Micromeritics, Norcross, GA, USA).

### 3.3. Preparation of RCC3-C10@silica

**Figure 6:**
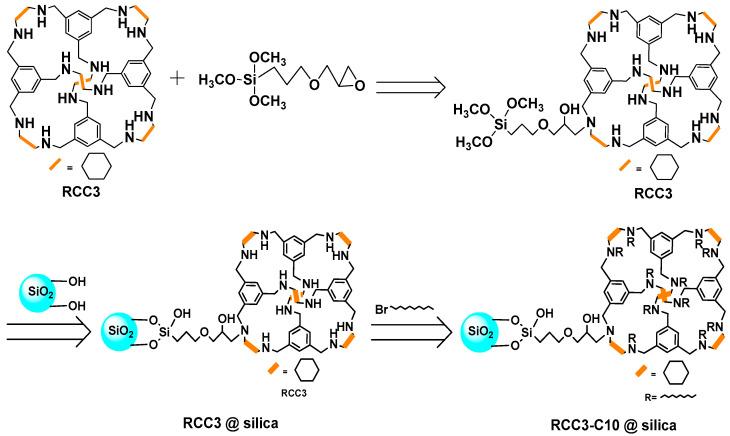
Preparation process of RCC3-C10@silica stationary phase.

POCs of CC3 [8] and RCC3 [29] crystals were synthesized according to the published literature by Andrew I. Cooper (Figure S1) The synthesis process of RCC3-C10@silica is shown in Figure 6. First, the RCC3 molecular cage was dissolved in toluene, then 3-(2,3-glycidoxy)propyltrimethoxysilane was added, and the reaction was carried out at 60 °C for 24 h. Then, the silica gel activated by hydrochloric acid was added, and the reaction was carried out at 110 °C for 24 h. The product was filtered through a sand core funnel. The silica gel molecular cage was dispersed in dry pyridine, and excess C10-Br was added. The reaction was heated at 80 °C for 24 h. After cooling to room temperature, the product was filtered and washed with absolute ethanol. After vacuum drying at 40 °C for 12 h, the RCC3-C10@silica molecular cage stationary phase was finally obtained.

### 3.4. Column Packing

An amount of 2.5 g RCC3-C10@silica was put in a beaker. The carbon tetrachloride was used as the homogenate. The methanol was used as the displacement solution. The RCC3-C10@silica was loaded into a stainless steel chromatographic column (4.6 mm × 150 mm) through a column packing machine at 40 MPa.

### 3.5. Sample Preparation and Chromatographic Conditions

Individual test analytes were dissolved in ethanol or methanol to make stock solutions and stored at 4 °C until use. The stock solution was then diluted to the desired concentration with the appropriate mobile phase to obtain dilutions for HPLC analysis. All chromatographic analyses were performed on SHIMADZU series high performance liquid chromatography systems. Chromatographic separation was performed at room temperature with a flow rate of 1.0 mL/min. The mobile phase was filtered through a 0.45 μm membrane before use.

## 4. Conclusions

In this paper, a C10-modified RCC3 molecular cage was covalently bonded to the surface of activated silica gel to prepare a multi-functional HPLC stationary phase RCC3-C10@silica, which exhibited good separation performance for 70 chiral and achiral compounds. The RCC3 and C10 groups in the newly prepared stationary phase framework provide a variety of interactions including hydrophobic interaction, π-π interaction, hydrogen bonding, steric interaction and the synergistic interaction of various forces, improving the RCC3-C10@silica stationary phase to exhibit good separation and analysis performance of Tanaka test mixtures, alkylbenzenes, PAHs, phenols, anilines, sulfonamides, nucleosides and flavonoids. The multiple chiral binding sites provided by the chiral stereo structure, based on the molecular cage skeleton, enhances the stationary phase to have good chiral resolution performance for the phenethyl alcohol, 1-phenyl-2-propanol, p-methyl Enantiomers of phenethyl alcohol and 1-phenyl-1-propanol. In addition, the RCC3-C10@silica molecular cage column has good column efficiency and reproducibility. In conclusion, the RCC3-C10@silica molecular cage stationary phase has multiple mechanisms of interactions in the RPLC mode and fully exhibits excellent multifunctional separation performance for chiral and achiral compounds. 

## Figures and Tables

**Table 1:** Chromatographic parameters of chiral drugs on the RCC3-C10@silica molecular cage column.

Solute	k_1_	k_2_	α	R_s_
phenyl ethanol	1.32	2.19	1.66	2.95
1-phenyl-2-propanol	1.85	3.07	1.66	3.73
p-methyl phenyl-ethanol	2.34	3.58	1.53	3.27
1-phenyl-1-propanol	2.64	4.45	1.69	4.09

## Data Availability

The datasets generated during and/or analyzed during the current study are available from the corresponding author on reasonable request.

## Supplementary Materials

The following supporting information can be downloaded at https://www.mdpi.com/article/10.3390/molecules27248895/s1. Figure S1: Synthesis of RCC3; Figure S2: FTIR of the silica gel, RCC3@silica, RCC3-C10@silica; Figure S3: TGA, DSC and DTG curves of RCC3-C10@silica stationary phase; Figure S4: Nitrogen adsorption and desorption isotherms on silica gel (a) and RCC3-C10@silica (b); Figure S5: Chromatograms of the Tananka test analytes on the RCC3-C10@silica molecular cage column and WondaSil^®^ C18-WR column; Figure S6: Plot of log k versus methylene number for different mobile phase compositions; Figure S7: Chromatograms of alkyl phenyl cyclic compounds on the RCC3-C10@silica molecular cage column and WondaSil^®^ C18-WR column; Figure S8: Plot of log k versus volume fraction of methanol and acetonitrile in mobile phase; Figure S9: Chromatograms of PAHs on the RCC3-C10@silica molecular cage column and WondaSil^®^ C18-WR column; Figure S10: Chromatograms of PAHs on the RCC3-C10@silica molecular cage column and WondaSil^®^ C18-WR column; Figure S11: Chromatograms of phenol compounds on the RCC3-C10@silica column and WondaSil^®^ C18-WR column; Figure S12: Chromatogram of nucleoside on the RCC3-C10@silica column and WondaSil^®^ C18-WR column; Figure S13: Chromatogram of sulfonamides on the RCC3-C10@silica column and WondaSil^®^ C18-WR column; Figure S14: Chromatogram of flavanones on the RCC3-C10@silica column and WondaSil^®^ C18-WR column; Figure S15: Repeatability of six compounds on RCC3-C10@silica column; Table S1: Retention factors and selection factors of Tanaka test analytes on RCC3-C10@silica column and WondaSil^®^ C18-WR column; Table S2: Retention factors and selection factors of PAHs on the RCC3-C10@silica molecular cage column and WondaSil^®^ C18-WR column; Table S3: Retention factors and selection factors of polar aromatic analytes on the RCC3-C10@silica column and WondaSil^®^ C18-WR column.
