# Supplementary material for: Porous Organic Cage-Embedded C10-Modified Silica as HPLC Stationary Phase and Its Multiple Separation Functions
Source: Molecules. 2022 Dec 14;27(24):8895. doi: 10.3390/molecules27248895 (PMC9782137; doi:10.3390/molecules27248895)
Supplement: Supplementary file 1 [file molecules-27-08895-s001.zip › molecules-2065091-supplementary.pdf]

Supporting Information

Porous Organic Cage-Embedded C10-Modified Silica as HPLC Stationary Phase and Its Multiple Separation Functions

Litao Wang [1], Siqi Han [1], Haiyang Yu [1], Qinghua Yu [1,2], Dong Pei [3], Wenjing Lv [1], Jiasheng Wang [1], Xingyu Li [1], Ruifang Ding [1,*], Qibao Wang [1,*] and Mei Lv [1,*]

1 School of Pharmacy, Jining Medical University, Jining 272000, China
2 School of Pharmacy, Weifang Medical University, Weifang 261000, China
3 Qingdao Center of Resource Chemistry & New Materials, Lanzhou Institute of Chemical Physics, Chinese Academy of Sciences, Qingdao 266000, China
*Correspondence: lvmei57010@163.com (M.L.); wqb_wangqibao@163.com (Q.W.); ruifangding@sina.com.cn (R.D.)

Table of Contents

1. Synthesis of RCC3…………………………...…………………………..…………..2

2. Characterization of RCC3-C10@silica……………...…......…………..……....…3

3. The chromatographic data of RCC3-C10@silica stationary phase…..…………....6

1. Synthesis of RCC3

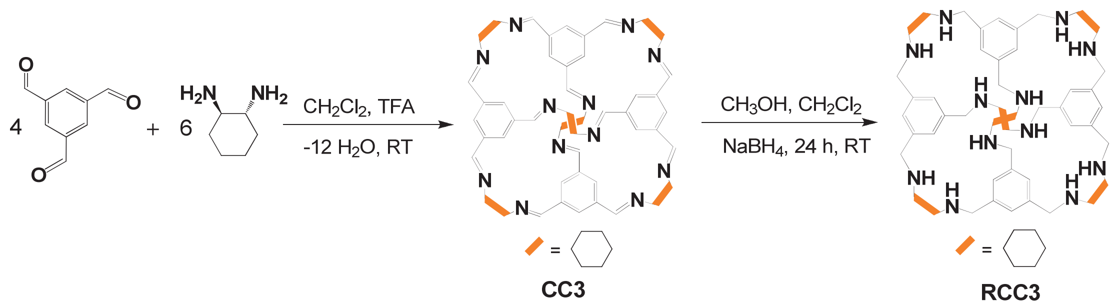

Figure S1. Synthesis of RCC3

.

2. Characterization of RCC3-C10@silica

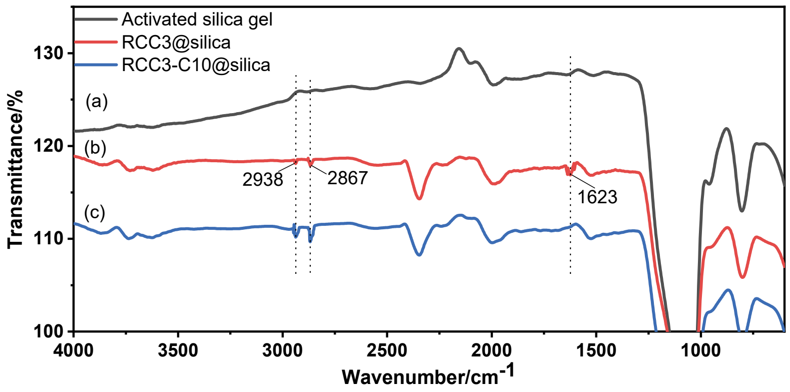

Figure S2. FTIR of the silica gel, RCC3@silica, RCC3-C10@silica.

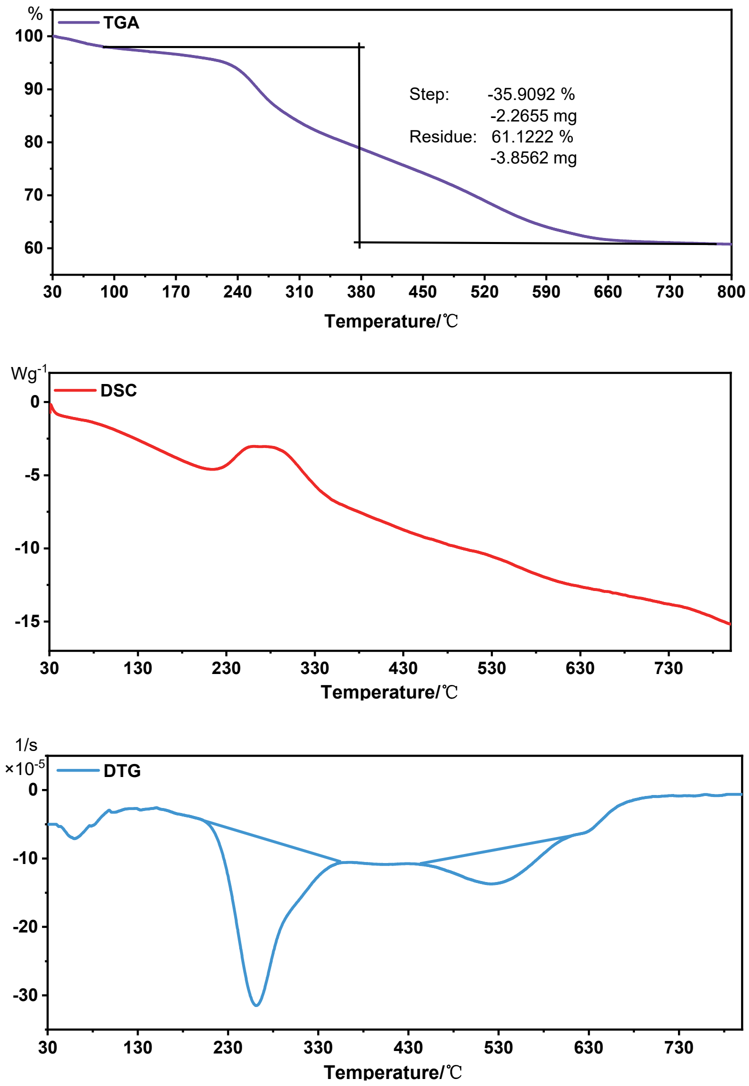

Figure S3. TGA, DSC and DTG curves of RCC3-C10@silica stationary phase.

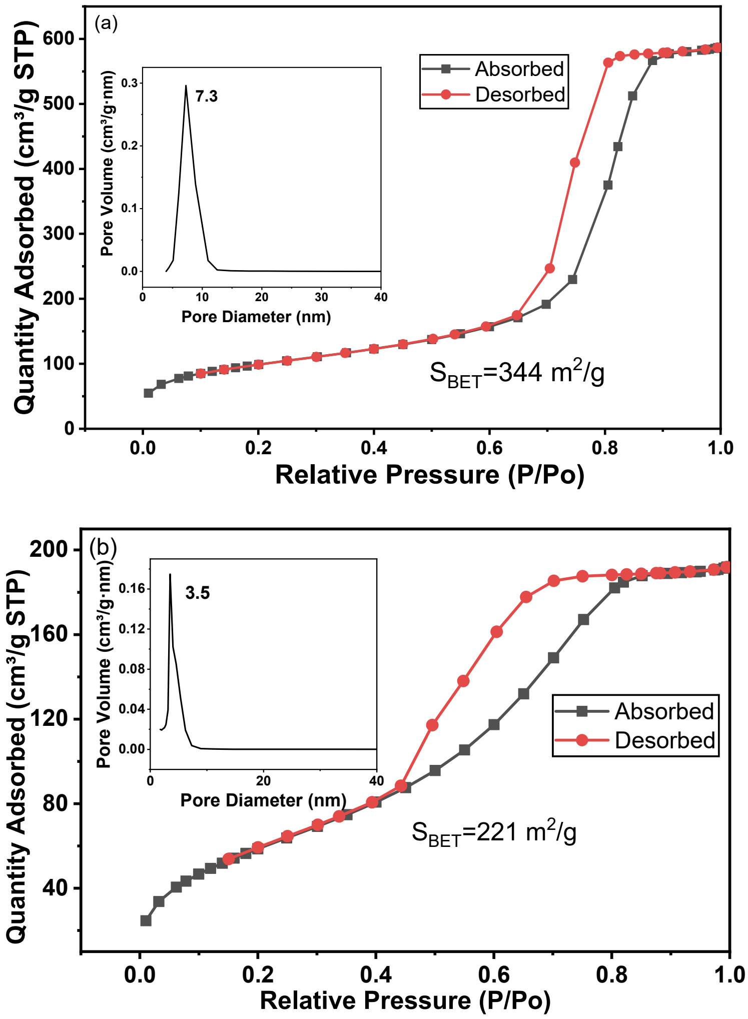

Figure S4. Nitrogen adsorption and desorption isotherms on silica gel (a) and RCC3-C10@silica (b).

3. The chromatographic data of RCC3-C10@silica stationary phase

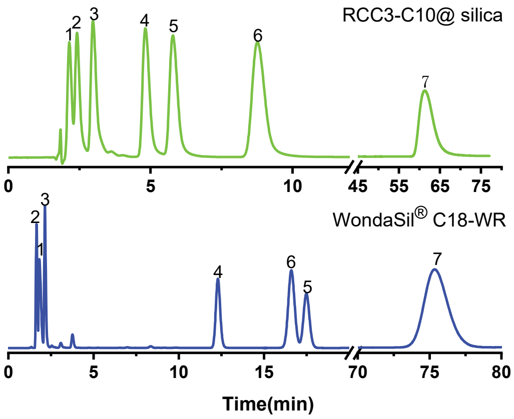

Figure S5. Chromatograms of the Tananka test analytes on the RCC3-C10@silica molecular cage column and WondaSil® C18-WR column. Analytes: (1) caffeine, (2) uracil, (3) phenol, (4) butylbenzene, (5) pentyl benzene, (6) o-terphenyl, (7) triphenylene. The mobile phase: 80% methanol/water (v/v); Flow rate: 1.0 ml/min; UV: 254 nm.

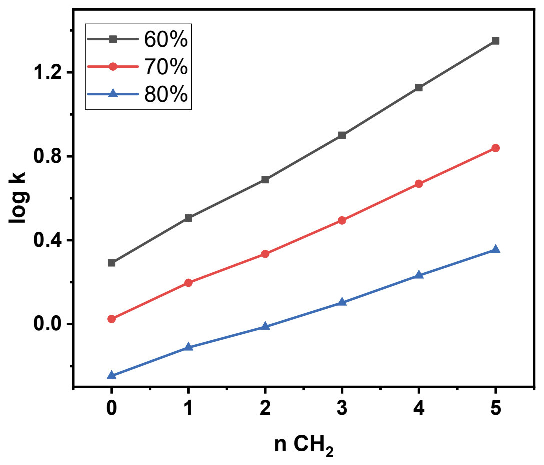

Figure S6. Plot of log k versus methylene number for different mobile phase compositions.

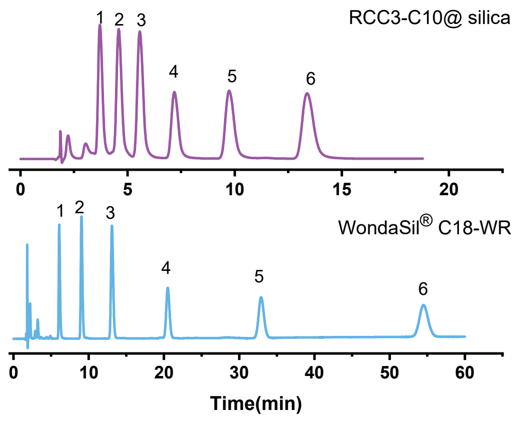

Figure S7. Chromatograms of alkyl phenyl cyclic compounds on the RCC3-C10@silica molecular cage column and WondaSil® C18-WR column. Analytes: (1) benzene, (2) toluene, (3) ethylbenzene, (4) propyl benzene, (5) butylbenzene, (6) pentyl benzene. Mobile phase: 70% methanol/water (v/v); Flow rate: 1.0 mL/min; UV: 254 nm.

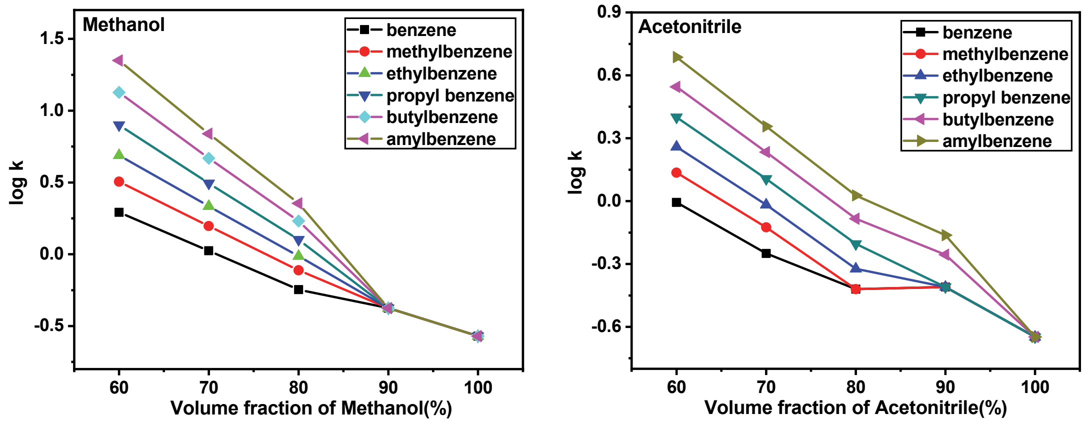

Figure S8. Plot of log k versus volume fraction of methanol and acetonitrile in mobile phase.

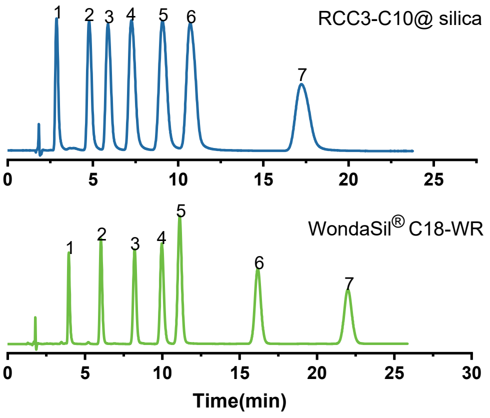

Figure S9. Chromatograms of PAHs on the RCC3-C10@silica molecular cage column and WondaSil® C18-WR column. Analytes: (1) benzene, (2) naphthalene, (3) biphenyl, (4) acenaphthene, (5) o-terphenyl, (6) anthracene, (7) m-terphenyl. Mobile phase: 80% methanol/water (v/v); Flow rate: 1.0 ml/min; UV: 254 nm.

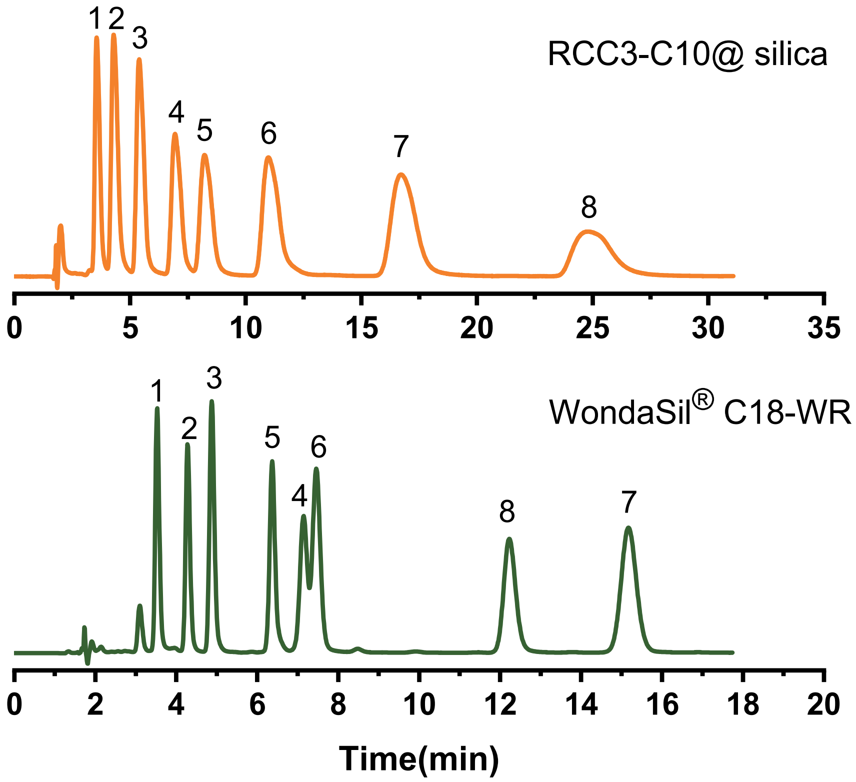

Figure S10. Chromatograms of PAHs on the RCC3-C10@silica molecular cage column and WondaSil® C18-WR column. Analytes: (1) 2-methylnaphthalene, (2) anthranone, (3) biphenyl (4) p-terphenyl, (5) pyrene, (6) chrysene, (7) triphenylene, (8) dibenz[a,h]anthracene. Mobile phase: 90% methanol/water (v/v); Flow rate: 1.0 ml/min; UV: 254 nm.

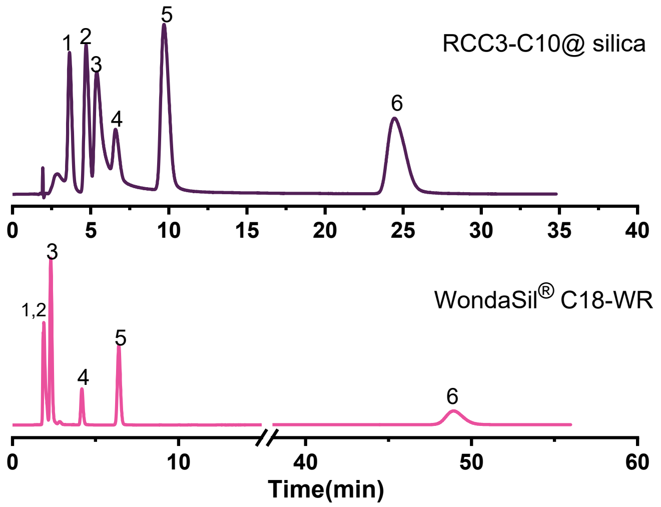

Figure S11. Chromatograms of phenol compounds on the RCC3-C10@silica column and WondaSil® C18-WR column. Analytes: (1) hydroquinone, (2) resorcinol, (3) catechol, (4) p-methyl phenol, (5) p-dimethyl phenol, (6) diphenyl ether. Mobile phase : 60% methanol/water (v/v). Flow rate: 1.0 ml/min; UV: 254 nm.

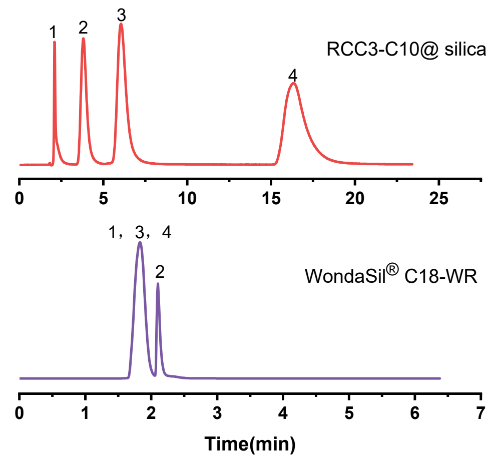

Figure S12. Chromatogram of nucleoside on the RCC3-C10@silica column and WondaSil® C18-WR column. Analytes: (1) cytidine, (2) thymidine, (3) guanosine, (4) inosine. Mobile phase: 30% methanol/ water; Flow rate: 1.0 mL/min; UV: 254 nm.

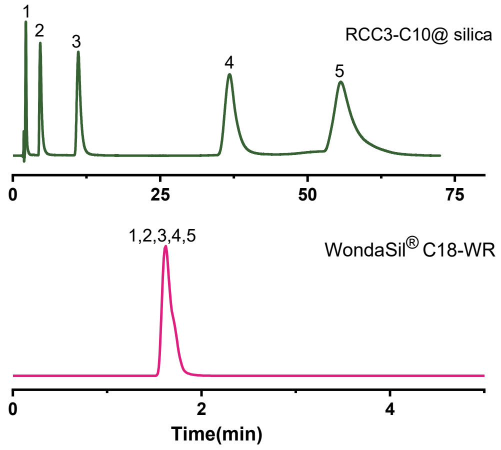

Figure S13. Chromatogram of sulfonamides on the RCC3-C10@silica column and

WondaSil® C18-WR column. Analytes: (1) sulfadiazine, (2) sulfadiazine, (3) sulfadiazine, (4) sulfadiazine, (5) sulfathiazole. Mobile phase: 80% methanol/water (v/v); Flow rate: 1.0 mL/min; UV: 254 nm.

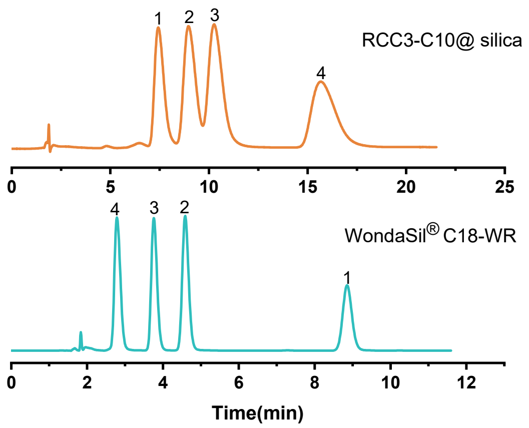

Figure S14. Chromatogram of flavanones on the RCC3-C10@silica column and WondaSil® C18-WR column. Analytes: (1) flavanones, (2) 6-hydroxyflavanones, (3) 4-hydroxyflavanones, (4) equol. Mobile phase: 70% methanol/water (v/v); Flow rate: 1.0 mL/min; UV: 254 nm.

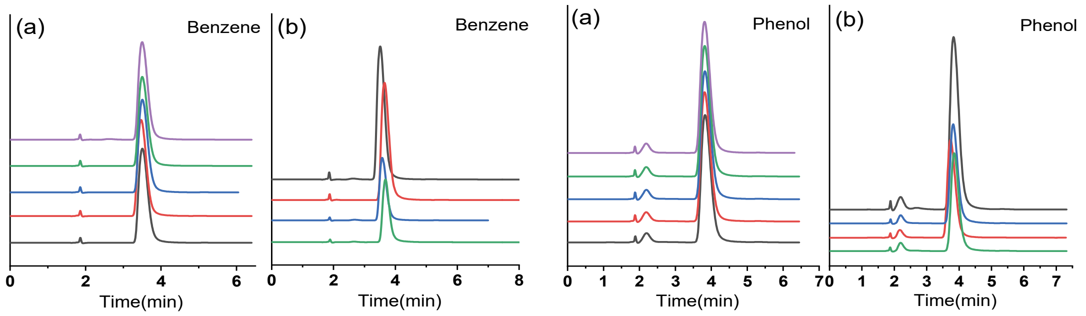

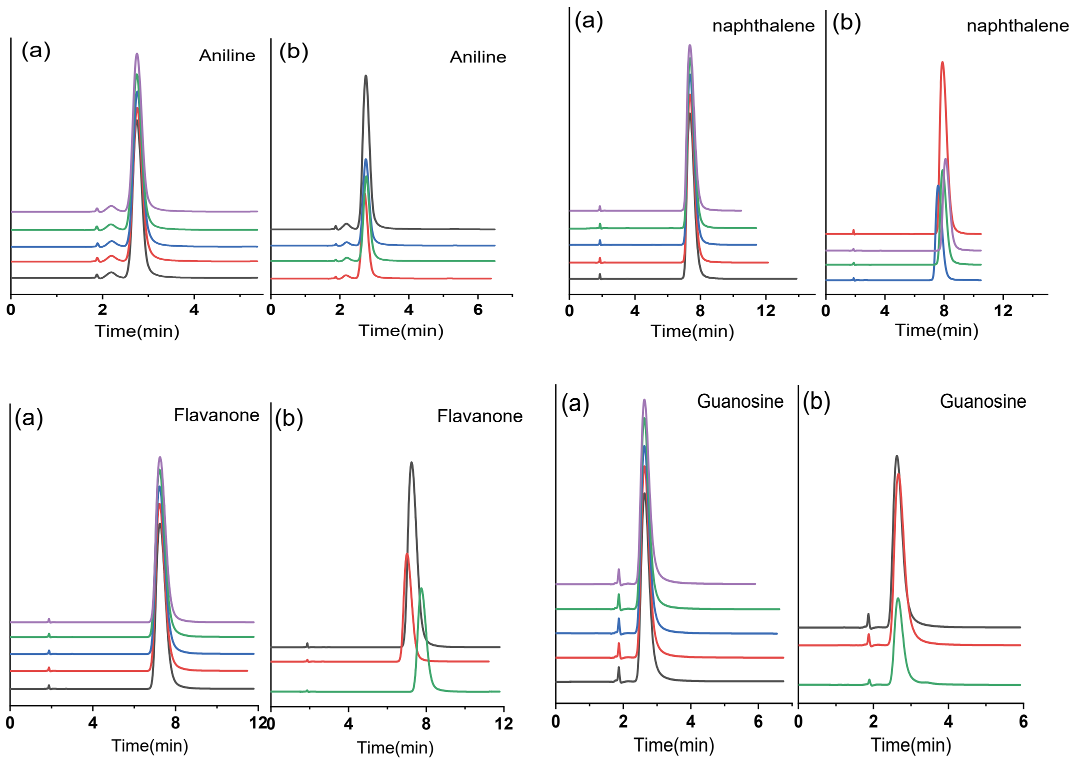

Figure S15. Repeatability of six compounds on RCC3-C10@silica column. (a) Intraday RSD of analyte, (b) Interday RSD of analyte.

Table S1. Retention factors and selection factors of Tanaka test analytes on RCC3-C10@silica column and WondaSil® C18-WR column.

Solute	RCC3-C10@silica		WondaSil® C18-WR	
	k	α	k	α
caffeine	0.17	1	0.35	1
uracil	0.32	1.88	0.23	0.66
phenol	0.62	3.65	0.59	1.68
butylbenzene	1.63	1	8.11	1
amylbenzene	2.16	1.32	11.95	1.47
o-terphenyl	3.79	1	11.30	1
triphenylene	32.50	8.58	54.81	4.85

Table S2. Retention factors and selection factors of PAHs on the RCC3-C10@silica molecular cage column and WondaSil® C18-WR column.

Solute	RCC3-C10@silica		WondaSil® C18-WR	
	k	α	k	α
2-methylnaphthalene	1.01	1	1.04	1
anthranone	1.42	1.40	1.47	1.42
p-Terphenyl	2.04	1.44	1.82	1.24
pyrene	2.93	1.44	3.13	1.72
chrysene	3.64	1.24	2.68	0.86
triphenylene	5.20	2.55	3.31	1.82
1, 2:5, 6-dibenzanthracene	8.46	1.63	7.76	2.34

Table S3. Retention factors and selection factors of polar aromatic analytes on the RCC3-C10@silica column and WondaSil® C18-WR column.

Solute	RCC3-C10@silica		WondaSil® C18-WR	
	k	α	k	α
p-aminophenol	0.27	1	0.42	1
m-aminophenol	0.53	1.96	0.42	1
o-aminophenol	0.84	1.58	0.64	1.52
phenol	1.72	2.05	1.40	2.19
hydroquinone	0.87	1	0.12	1
resorcinol	1.41	1.62	0.21	1.75
catechol	1.78	1.26	0.37	3.08
p-methyl phenol	2.37	1	1.48	1
p-dimethyl phenol	3.96	1.67	2.80	1.89
diphenyl ether	11.6	2.93	28.03	10.01
3, 5-dimethyl phenol	3.70	1	3.55	1
p-tert-butyl phenol	6.94	1.88	8.59	2.42
2-naphthol	9.02	1.30	3.81	0.44
p-phenylenediamine	0.76	1	0.09	1
m-phenylenediamine	1.06	1.39	0.09	1
o-phenylenediamine	1.45	1.37	0.24	2.67
aniline	2.07	1.43	0.68	2.83
p-toluidine	2.56	1	1.18	1
3, 4-dimethylamine	3.35	1.31	1.86	1.58
4, 4-biphenylenediamine	4.95	1.48	0.36	0.19
m-nitroaniline	6.07	1	0.95	1
o-nitroaniline	7.62	1.26	1.05	1.10
